# Treatment-induced stemness and lineage plasticity in driving prostate cancer therapy resistance

**DOI:** 10.47248/chp2401010005

**Published:** 2024-08-25

**Authors:** Anmbreen Jamroze, Xiaozhuo Liu, Dean G. Tang

**Affiliations:** 1. Department of Pharmacology & Therapeutics, Roswell Park Comprehensive Cancer Center, Buffalo, NY 14263, USA; 2. Experimental Therapeutics (ET) Graduate Program, University at Buffalo & Roswell Park Comprehensive Cancer Center, NY 14263, USA

**Keywords:** stemness, lineage plasticity, androgen receptor, prostate cancer, cancer cell heterogeneity, cancer stem cells, therapy resistance, castration-resistant prostate cancer

## Abstract

Most human cancers are heterogeneous consisting of cancer cells at different epigenetic and transcriptional states and with distinct phenotypes, functions, and drug sensitivities. This inherent cancer cell heterogeneity contributes to tumor resistance to clinical treatment, especially the molecularly targeted therapies such as tyrosine kinase inhibitors (TKIs) and androgen receptor signaling inhibitors (ARSIs). Therapeutic interventions, in turn, induce lineage plasticity (also called lineage infidelity) in cancer cells that also drives therapy resistance. In this Perspective, we focus our discussions on cancer cell lineage plasticity manifested as treatment-induced switching of epithelial cancer cells to basal/stem-like, mesenchymal, and neural lineages. We employ prostate cancer (PCa) as the prime example to highlight ARSI-induced lineage plasticity during and towards development of castration-resistant PCa (CRPC). We further discuss how the tumor microenvironment (TME) influences therapy-induced lineage plasticity. Finally, we offer an updated summary on the regulators and mechanisms driving cancer cell lineage infidelity, which should be therapeutically targeted to extend the therapeutic window and improve patients’ survival.

## 1. Introduction: A general discussion on cancer stemness and plasticity

Therapy resistance remains a significant challenge in cancer treatment, leading to poor outcomes and limited therapeutic options for patients. Understanding the underlying mechanisms of therapy resistance is crucial for improving treatment strategies and patient outcomes. One emerging area of research focuses on therapy-induced stemness and lineage plasticity, which have been implicated as key factors in mediating and driving therapy resistance. Therapy-induced stemness refers to the acquisition of stem cell gene expression profiles and functional properties by cancer cells following exposure to therapies. These stemness traits, such as self-renewal and multipotency, allow cancer cells to persist, regenerate and develop resistance to treatment. Multiple factors contribute to therapy-induced stemness, including genetic and epigenetic alterations, activation of signaling pathways, and interactions with the tumor microenvironment.

**Figure 1:**
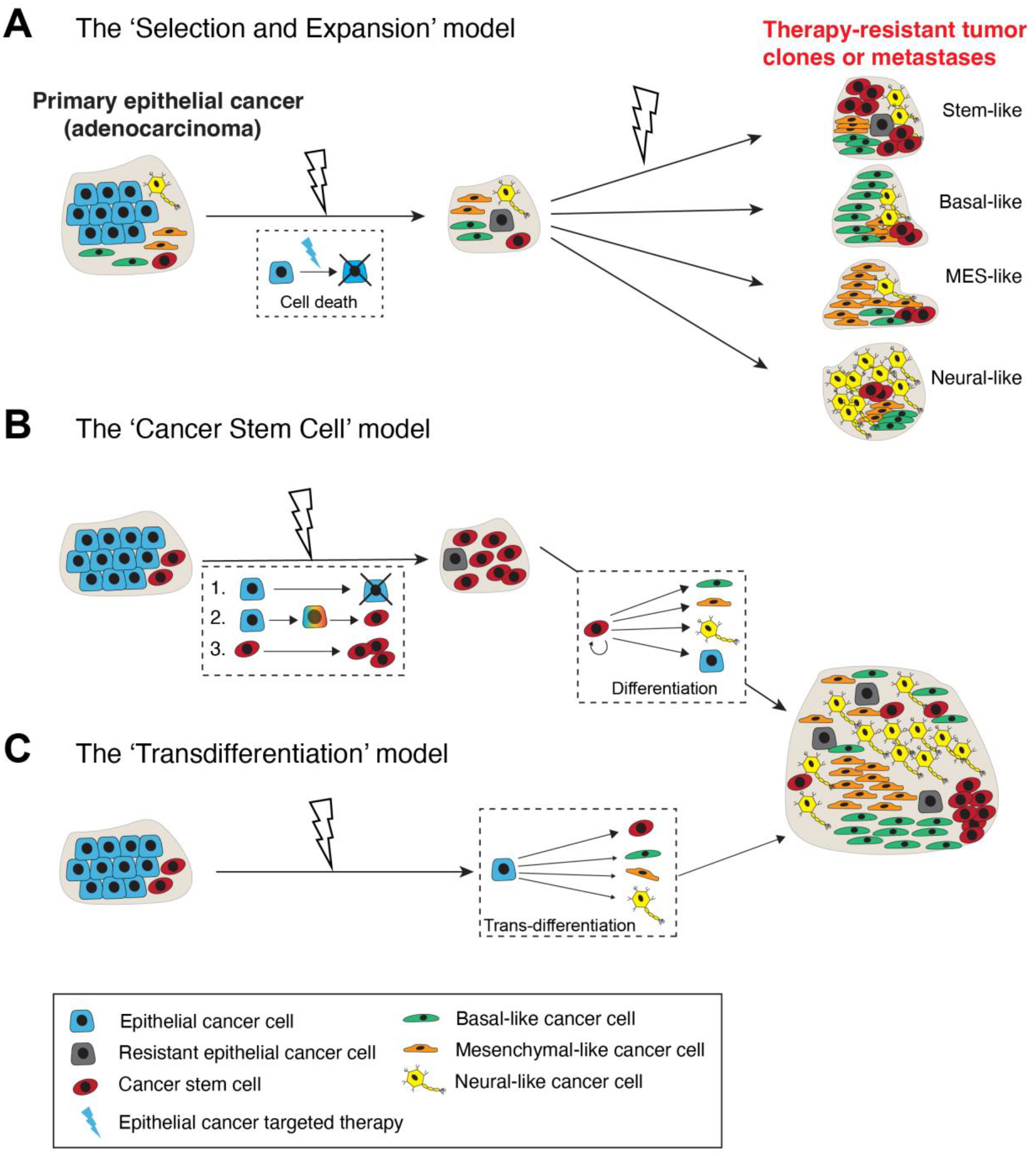
Three models of generating lineage-distinct and therapy-resistant tumors from an adenocarcinoma. (**A**) The lineage-specific ‘Selection and Expansion’ model. The depicted adenocarcinoma consists of bulk epithelial cancer cells as well as minor subsets of stem-, basal-, mesenchymal- (MES) and neural-like cancer cells, the latter of which may become selected for and expanded during targeted therapies leading to therapy-resistant tumors that are enriched in stem-, basal-, MES- or neural-lineage features. (**B**) The ‘Cancer Stem Cell’ model. Targeted therapies lead to the death of (target-expressing) epithelial cancer cells (cell fate 1) and enrichment of cancer stem cells (CSCs) because of therapy-induced de-differentiation (reprogramming) of bulk epithelial cancer cells to CSCs (cell fate 2) or the expansion of pre-existing stem-like cancer cells (cell fate 3). CSCs then undergo ‘multi-lineage’ differentiation to give rise to stem-, basal-, MES- or neural-like endpoint tumors. (**C**) The ‘Transdifferentiation’ model. Bulk epithelial cancer cells in the depicted adenocarcinoma are directly converted by therapies (or genetic/epigenetic alterations) to different lineages without going through an intermediate state.

Cancer cells are highly plastic with respect to their phenotypes, transcriptomes, epigenome (cell state) and functions. Cancer cell plasticity encompasses cellular and lineage plasticity: cellular plasticity refers to phenotypic and functional changes of cancer cells along the same ‘developmental’ lineage whereas lineage plasticity involves the ability of cancer cells to undergo phenotypic changes and switch between different cellular lineages. At least 3 conceptual models can explain the cellular mechanisms underpinning lineage plasticity, which allows cancer cells to adopt alternative cell fates that are more resistant to therapy (Figure 1; see below). Cancer cells may transition from a differentiated state to a more stem cell-like state or switch to a different lineage altogether. The transition of tumor cells into a distinct histological subtype has been linked to a reduced dependency on the initial oncogenic driver, leading to therapeutic resistance. An illustrative example of lineage plasticity in cancer is the transformation of adenocarcinomas into aggressive neuroendocrine derivatives. Frequently observed in lung cancers with an EGFR mutation, this phenomenon has been identified in various adenocarcinomas, such as prostate cancer (PCa) under the influence of antiandrogens (see below). The practice of re-biopsying tumors upon disease progression has contributed to recognizing these resistance mechanisms and enhancing our comprehension of their underlying biological processes.

The concept of cancer cell plasticity is not new and was reported decades ago. Take PCa, histological analysis of patient tumor sections nearly 30 years ago [1] revealed that a subset of basal cells in benign glands frequently co-expresses both basal cell-specific cytokeratins and luminal differentiation marker PSA (prostate-specific antigen) whereas some PCa cells are phenotypically amphicrine expressing both PSA (*i.e*., exocrine) and chromogranins (endocrine). These histological observations imply multi-lineage differentiation and/or lineage plasticity in benign prostatic glands and PCa. It was also reported, about 30 years, that cultured LNCaP cells, the most widely used PCa cell line, could switch the lineage and undergo ‘terminal’ neuroendocrine (NE) differentiation under the influence of increased intracellular cAMP [2]. Subsequent studies in genetically engineered mouse models (GEMMs) of PCa indeed revealed significant developmental plasticity in normal mouse prostate basal cells [3] and demonstrated that deletion of tumor suppressor genes *Pten* and *Trp53* in luminal epithelial cells led to epithelial-to-mesenchymal transition (EMT) and development of aggressive PCa [4]. In 2017, three papers, two in GEMMs [5,6] and one using human PCa cells [7], revived the field of studies on PCa lineage plasticity. By now, lineage plasticity has been widely reported in multiple cancer types and many excellent reviews on the topic have been published [8–19]. A remarkable example is the histological transformation of epithelial non-small cell lung cancer (NSCLC) to small cell lung cancer (SCLC) upon treatment by tyrosine kinase inhibitors (TKIs) or immunotherapies [18]. Moreover, SCLC display cancer cell plasticity and phenotypic switching between subtype states, defined by different lineage-related molecular subsets [14].

Cancer therapies, especially targeted cancer drugs, may drive an epithelial adenocarcinoma to a therapy-resistant tumor subtype that is stem cell-enriched and exhibits a different lineage resembling basal, mesenchymal (MES) or neural cells via at least 3 cellular mechanisms (Figure 1). The first mechanism invokes a *direct* lineage-specific ‘Selection and Expansion’ model (Figure 1A). As pre-treatment epithelial cancer is never homogeneous but rather heterogeneous consisting of both bulk epithelial cancer cells as well as minor subpopulations of cancer cells already possessing stem/basal/MES/neural cell phenotypes and properties, it is conceptually possible that these pre-existing non-epithelial cancer cell populations become selected for and (clonally) expand during the long course of clinical therapy leading to heterogeneous tumor cell clones and metastases that are therapy-resistant (Figure 1A). Alternatively, as treatment-naïve tumors harbor a functional subpopulation of stem-like cancer cells called cancer stem cells (CSCs), which are generally immature and frequently lack the expression of therapeutic target such as androgen receptor (AR) in PCa, targeted therapies would preferentially eliminate target-positive cells and enrich target-negative CSCs, which could subsequently differentiate along other lineages (Figure 1B; the ‘Cancer Stem Cell’ model; [15,20]). Finally, bulk epithelial cancer cells could, potentially, be directly converted, via a process called ‘transdifferentiation’ or reprogramming, to a non-epithelial lineage in the endpoint tumors (Figure 1C). Studies in many cancers, e.g., genetic loss of tumor suppressors *Pten*, *Rb1* and/or *Trp53* in PCa [4–7], also support this route to lineage plasticity and as a mechanism of therapy resistance.

## 2. Lineage plasticity associated with treatment and tumor progression drives therapy resistance: A general phenomenon in multiple cancers

Treatment- and mutation-induced lineage plasticity in driving therapy resistance has been reported in many cancers [8–19], especially in PCa (see the Section below) and lung cancer. Lung cancer, the leading cause of cancer-related mortality in both men and women, encompasses diverse subtypes, including epithelial NSCLC and the NE subtype SCLC. Transition from NSCLC to SCLC histology has been reported as a mechanism of treatment resistance in patients who received TKIs targeting EGFR, ALK and ROS1. The mechanism could arise from therapy-induced lineage plasticity and/or clonal selection of pre-existing SCLC cells [12,14,18,19]. In lung adenocarcinoma (LUAD), cancer cell identity is governed by lineage-specifying TFs FOXA1 and FOXA2, whose loss triggers the collapse of a dual-identity state associated with lung adenocarcinoma progression, leading to alternative cellular identities linked to non-proliferative states [21]. EGFR-targeting TKIs can drive resistance in EGFR-mutated NSCLC by causing the loss of adenocarcinoma lineage genes, and genetic alterations, immune microenvironment, and T790M status all contribute to divergent TKI resistance trajectories and lineage transformation to SCLC [22]. SCLC also comprises multiple molecular subtypes, associated with distinct responses to different therapies and defined by the expression of MYC family members and lineage-related TFs such as ASCL1, NEUROD1, and POU2F3 [14]. Moreover, SCLC exhibits a high degree of intratumoral heterogeneity, with recent studies suggesting the existence of tumor cell plasticity and phenotypic switching between subtypes [14]. Single-cell transcriptomic profiling of genetically engineered mouse lung tumors at seven stages, from pre-neoplastic hyperplasia to adenocarcinoma, revealed that cancer cells progressively adopt alternate lineage identities, computationally predicted to be mediated through a common transitional, high-plasticity cell state where cells display high capacity for differentiation, proliferation, and chemoresistance [23]. Detailed genomic, epigenomic, transcriptomic, and proteome characterization of combined LUAD/SCLC tumors, as well as pre-/post-transformation samples, supports that the NE transformation is primarily driven by transcriptional reprogramming rather than mutational events, with enhanced expression of genes involved in PRC2, PI3K/AKT and NOTCH pathways [24]. By contrast, primary LUAD are characterized by the emergence of regenerative cell types typically seen in response to lung injury, with reactivation of endoderm and lung-specifying TFs such as SOX2 and SOX9 that recapitulate more primitive transcriptional programs [25].

Something analogous has been observed in pancreatic ductal adenocarcinoma (PDAC), where transcriptional regulatory networks essential for proper lineage specification and differentiation during pancreas development are reactivated or become deregulated in the context of cancer and exacerbate progression towards an aggressive phenotype [26]. Similarly, bladder cancers originating from the urothelium often exhibit lineage plasticity with regions of urothelial carcinoma adjacent to or admixed with regions of divergent histomorphology, most commonly squamous differentiation [27]. Lineage plasticity in bladder cancers with squamous differentiation was associated with the loss of expression of crucial TFs including FOXA1, GATA3, and PPAR_γ_, which are pivotal for maintaining urothelial cell identity [27].

ARID1A, a subunit of the SWI/SNF chromatin remodeling complex, is commonly mutated in endocrine-resistant estrogen receptor (ER) positive breast cancer, and its inactivation leads to resistance to ER degraders by facilitating a switch from ER-dependent luminal cells to ER-independent basal-like cells [28]. Cellular plasticity is triggered by loss of ARID1A-dependent SWI/SNF complex targeting genomic sites of the luminal lineage-determining TFs including ER, FOXA1 and GATA3 [28]. In hepatocellular carcinoma (HCC), reactivation of CLDN6 (highly expressed in embryonic stem cells and markedly decreased in differentiated normal tissues) induced phenotypic shift from hepatic lineage to biliary lineage, which was more refractory to sorafenib treatment [29]. Tumor lineage plasticity and altered cellular identity were associated with and potentially induced by the CLDN6/TJP2 (tight junction protein 2)/YAP1 (Yes-associated protein 1) signaling axis [29].

Single-cell multi-omics analyses highlighted lineage plasticity also in *KMT2A*-rearranged infant acute lymphoblastic leukemia (ALL) patients of <6 months, and surprisingly, the steroid response pathways were downregulated in the most immature blasts from younger patients [30]. In melanoma, loss of SOX10 reduces proliferation, confers invasive properties via upregulating mesenchymal and extracellular matrix genes, and promotes tolerance to BRAF and/or MEK inhibitors, suggesting that SOX10 mediates phenotypic switching in cutaneous melanoma to produce a targeted inhibitor tolerant state that is likely a prelude to the acquisition of resistance [31].

In summary, lineage plasticity, associated with therapeutic pressure, tumor progression and TME changes, has emerged to be a central player in driving treatment resistance in a variety of tumors.

## 3. Androgen receptor (AR) heterogeneity and lineage plasticity in PCa: Essential contributors to castration-resistant PCa (CRPC)

**Figure 2:**
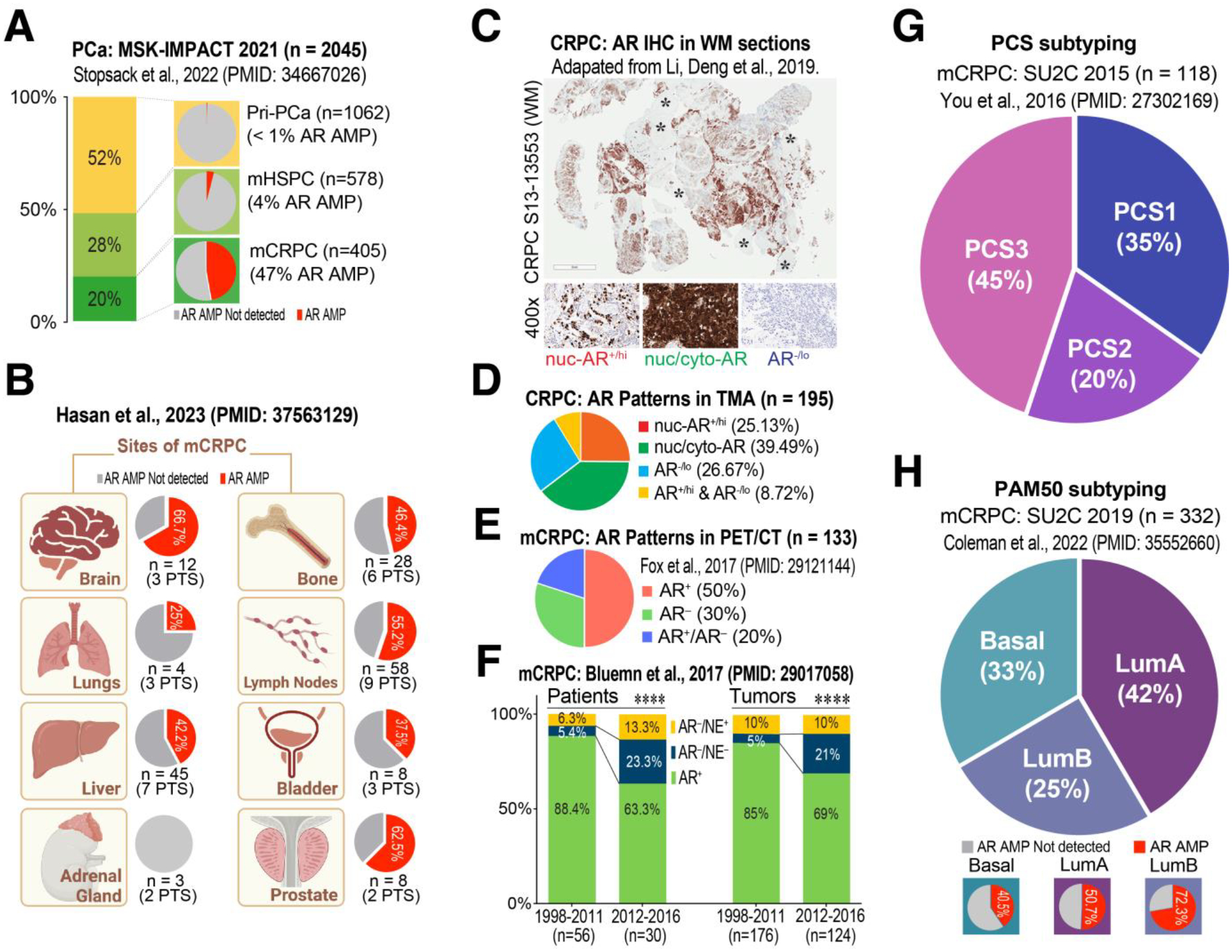
Heterogeneity in AR expression and CRPC subtypes. (**A**) Comparative analysis of *AR* amplification (AMP) status in primary PCa (Pri-PCa), metastatic hormone-sensitive PCa (mHSPC), and mCRPC. The percentages of *AR* AMP in each stage are indicated. The original data was retrieved from the MSK-IMPACT 2021 through cBioportal (https://www.cbioportal.org/) and Stopsack *et al*., 2022 (ref. [37]). The number of patients in each category is indicated in parentheses. (**B**) *AR* AMP status in different metastatic sites of mCRPC, with the percentages of *AR* AMP in each site indicated in the pie chart (Data source: Hasan *et al*., 2023; ref. [38]). The number of patients (PTS) and samples (n) analyzed for each site are indicated. (**C,D**) AR heterogeneity in CRPC. Shown are patterns of AR protein expression in CRPC tissue microarrays (TMA) consisting of 195 CRPC cores. C: AR IHC staining in whole-mount (WM) CRPC in the primary site (top; asterisks indicate AR^−/lo^ CRPC areas) and zoom-in images showing 3 distribution patterns of AR (bottom). D: Quantification of AR expression patterns in 195 CRPC cores. The IHC images in C were adapted, with permission, from Li, Deng et al., 2018 (ref. [33]). (**E**) AR heterogeneity in mCRPC lesions as determined by positron emission tomography/computed tomography (PET/CT) scans of 133 CRPC patients between 2007 and 2012 at the MSKCC. PET/CT imaging was conducted using fluoro-2-D-deoxyglucose F18 ([^18^F]-FDG) to map all glycolytic mCRPC lesions (*i.e*., the baseline) and the fluorodihydrotestosterone F18 ([^18^F]-FDHT) to map specifically the AR^+^ mCRPC lesions. Shown is the pie chart replotted from proportions of [^18^F]-FDHT-positive (AR^+^), [^18^F]-FDHT-negative/[^18^F]-FDG-positive (AR^−^), and mixed (AR^+^/AR^−^) phenotypes of the metastatic lesions. Data was replotted from Fox *et al*., 2017 (ref. [43]). (**F**) Significantly increased patients with AR^−^ mCRPC and significantly increased AR^−^ mCRPC tumors since the clinical use of next-generation ARSIs. Shown are the bar graphs presenting the % PCa patients with AR^+^ or AR^−^ (including both AR^−^/NE^+^ and AR^−^/NE^−^) mCRPC lesions (left) and the % metastatic tumors that were either AR^+^ or AR^−^ (including both AR^−^/NE^+^ and AR^−^/NE^−^) (right) in patient cohorts from the era prior to the approval of new ARSIs such as Enza and Abi (1998–2011) *vs*. the post-approval period (2012–2016). Chi-square tests indicate a significantly increased proportion of the AR^−^ subtypes (****, P < 0.0001). Data were replotted from Bluemn *et al*., 2017 (ref. [44]). (**G**) Transcriptional subtyping of mCRPC using the PCS system from the SU2C 2015 dataset. The percentages of PCS1, PCS2, and PCS3 subtypes are depicted in a pie chart. Data was replotted from You *et al*., 2016 (ref. [45]). (**H**) Transcriptional subtyping of mCRPC using the PAM50 system from the SU2C 2019 dataset. The proportions of LumA, LumB, and Basal subtypes are presented in a pie chart. Data was replotted from Coleman *et al*., 202 (ref. [46]).

**Figure 3:**
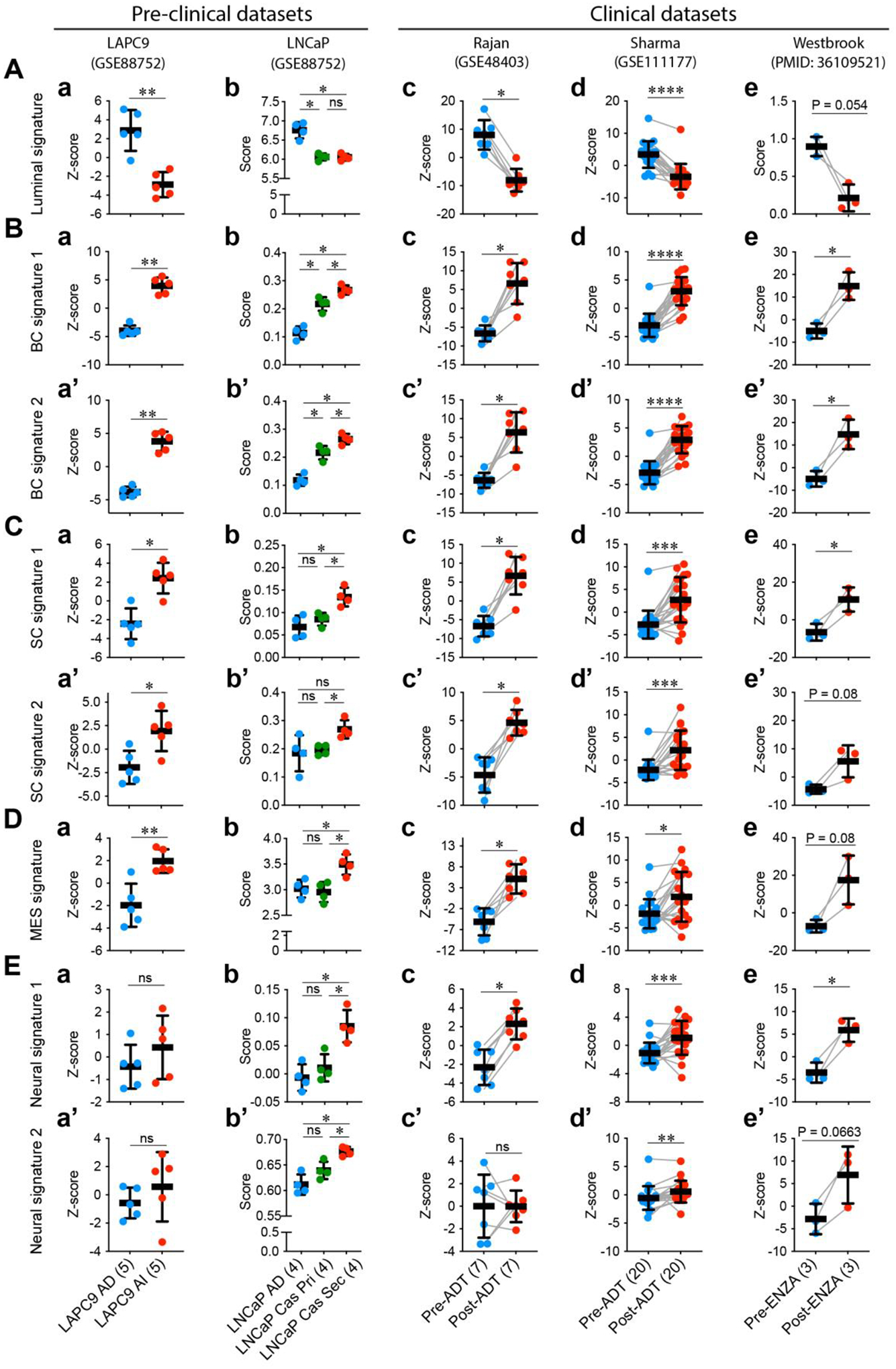
ARSI treatment induces PCa lineage plasticity in both preclinical and clinical datasets. Therapies targeting AR signaling axis lead to (**A**) loss of luminal cell identity (*i.e*., gene signature) but gain of basal-like (**B**), stem cell-like (**C**), MES-like (**D**), or neural-like (**E**) gene signatures in both experimental castration models (LAPC9 and LNCaP) and clinical patient samples including short-term (neoadjuvant) ADT (Rajan and Sharma) as well as the long-term Enza treatment (Westbrook). Gene signatures correlated with different cell types were obtained from the Molecular Signatures Database (MSigDB, ref. [87,88]) and the following references: (**A**) Luminal signature (396 genes; ref. [89]); (**B**) Basal cell (BC) signatures, including BC signature 1 (373 genes; ref. [89]) and BC signature 2 (56 genes; ref. [7]); (**C**) Stem Cell (SC) signatures, including SC signature 1 (480 genes; ref.[90]) and SC signature 2 (262 genes; ref. [90]); (**D**) MES signature (465 genes; ref. [91]); (**E**) Neural-like signatures, including Neural signature 1 (147 genes; ref. [92]) and Neural signature 2 (177 genes; ref. [93]). Transcriptome-based gene signature scores were generated based on the signature gene set expression values from the RNA-seq data downloaded from the following datasets: (**a,a’**) LAPC9, GSE88752 (ref. [33]); (**b,b’**) LNCaP, GSE88752 (ref. [33]); (**c,c’**) GSE48403 (ref. [95]); (**d,d’**) GSE111177 (ref. [96]); (**e,e’**) ref.[82]. Briefly, gene signature scores were calculated using a combined Z-score normalization method (ref. [97]) or single sample gene set enrichment analysis (ssGSEA) score method from the GSVA package (ref. [98]). Sample sizes are indicated. Within the plots, results are shown as mean ± SD. The pairs are linked with a grey line. Significance was calculated by two-tailed unpaired (a, b, a’, b’) or paired (c, d, e, c’, d’, e’) Student’s *t*-test (ns, not significant; *, P < 0.05; **, P < 0.01; ***, P < 0.001; ****, P < 0.0001).

The American Cancer Society estimates that ~35,000 American men will die from metastatic castration-resistant PCa (mCRPC) in 2024 [32]. PCa incidence rates, after 2 decades of decline, increased 3% per year from 2014–2019 driven by advanced PCa diagnosis. The proportion of men diagnosed with distant metastases, who have a 5-year survival rate of ~30%, has doubled from 2011 to 2019 when the next-generation Androgen Receptor Signaling Inhibitors or ARSIs (e.g., enzalutamide, Enza) were introduced to the clinic. Advanced and metastatic PCa patients are generally treated with androgen deprivation therapy (ADT) or a combination of ADT with one of the ARSIs including Enza, Abiraterone acetate (Abi), Apalutamide, and Darolutamide. Most treated patients unfortunately become refractory to ADT/ARSI within about 2 years. Although many molecular mechanisms have been reported to drive ADT/ARSI resistance, little is known about the population of tumor cells that survive these therapies and mediate PCa recurrence. Recent studies suggest that intrinsic PCa heterogeneity (Figure 2) and (treatment and genetic mutation) induced PCa cell plasticity (Figure 3) go hand-in-hand during tumor evolution and treatment response, driving ADT/ARSI resistance [15,20,33–35].

PCa is a hormone-driven tumor and most advanced PCa patients are ‘homogeneously’ treated by hormone blockade using inhibitors of testosterone production and AR signaling [15]. As ADT/ARSIs primarily target AR-expressing (AR^+^) PCa cells and interfere with AR signal transduction, one of the major molecular mechanisms driving ADT/ARSI resistance, understandably, involves the structural alterations of *AR* itself and expression of ligand-independent AR splice variants called AR-V’s [36]. This point can be readily appreciated by analyzing *AR* genomic amplifications (AMP) in 2,045 PCa cases in the MSK-IMPACT 2021 dataset consisting of 1,062 primary tumors, 578 metastatic hormone-sensitive PCa (mHSPC) and 405 mCRPC patients [37]: *AR* AMP was virtually non-existent in primary PCa and very low (4%) in mHSPC but was detected in 47% of mCRPC (Figure 2A), indicating that *AR* AMP is strictly treatment-induced! Similarly, a recent study [38] revealed a wide range of the prevalence of *AR* AMP (0–67%) in PCa metastasized to different end organs (Figure 2B).

Treatment-induced amplification of the therapeutic target, *i.e*., AR, is not the only problem, as treatment-naïve PCa is already heterogeneous with respect to AR expression such that there exist a population of AR non-expressing (AR^−/lo^) PCa cells [15,39–42]. These cells generally represent a small fraction (~10%) of the tumor bulk in treatment-naïve PCa but frequently become enriched in high-grade tumors. Expectedly, the pre-existing AR^−/lo^ PCa cells, characterized by inherently low AR activity [41], are much less responsive to ADT/ARSI [15,33] and often become a major cell population in mCRPC (Figure 2C–H). For instance, analyzing 195 CRPC specimens, Li *et al*. demonstrated that ~27% were AR^−/lo^ with another ~9% being mixed AR^+^/AR^−/lo^ [33] (Figure 2C,D). Fox *et al*. developed a dual PET/CT imaging strategy to analyze >2,000 mCRPC foci ([^18^F]-FDG positive) in 133 mCRPC patients and found that as much as 30% of the metastatic lesions were AR^−^, *i.e*., unable to bind the [^18^F]-DHT probe with another 20% of the lesions having the mixed AR^+^/AR^−^ phenotype [43] (Figure 2E). Similarly, a study from Bluemn *et al*. suggests that the % of patients with AR^−^ (including both AR^−^/NE^−^ and AR^−^/NE^+^) mCRPC were tripled (*i.e*., from about 10% to 36.6%) and the AR^−^ tumors were doubled (from 15% to 31%) in the contemporary era (2012–2016) following the widespread use of more effective ARSIs, Enza and Abi, compared to the period before their FDA approval (1998–2011) [44] (Figure 2F). These examples highlight the increase in AR^−/lo^ PCa cells and AR^−/lo^ mCRPC lesions induced by the new generation of powerful antiandrogens. (m)CRPC is also transcriptionally heterogeneous. For example, You *et al*. developed a transcriptome-based PCa Classification System (PCS) and found that the most aggressive mCRPC subtype, PCS1, has an AR-V7 signature [45] (Figure 2G). Similarly, Colemen *et al*. employed the PAM50 algorithm to classify the 332 mCRPC in SU2C 2019 to Basal, LumA and LumB subtypes that have different *AR* AMP and differential ADT responses and clinical outcomes [46] (Figure 2H).

In addition to heterogeneity in AR expression levels and patterns and ADT/ARSI-induced *AR* AMP, and increases in AR^−/lo^ mCRPC lesions and transcriptomic diversity in mCRPC, yet another problem is related to treatment-induced lineage plasticity in PCa cells. As mentioned earlier, the phenomenon of lineage plasticity in mediating therapy resistance in PCa was ‘rediscovered’ in 2017 by 3 independent studies [5–7]. Lineage plasticity in PCa refers to the ability of PCa cells to undergo histological transformation as an adaptive response to genetic mutations, environmental stimuli and anti-cancer therapy. Lineage plasticity associated with PCa progression and therapy resistance enables changes in the cancer cell’s identity and behavior, with the acquisition of characteristics associated with other cell lineages such as basal-, MES and/or, especially, neural lineages (Figure 1) [4–7,9,11,15,39,40,47–86]. In PCa, lineage plasticity has been mechanistically linked to genetic mutations [4–7,19], epigenetic alterations [11,12,15,17,28,30,54,78,85], inflammatory signaling [69–71], metabolic reprogramming [52,75,76] and therapeutic treatments including castration/antiandrogens [5–7,55,59,65,74,82] and DNA damage reagents [15,86]. Treatment-induced PCa lineage plasticity can be readily appreciated in both preclinical models as well as mCRPC patient tumors treated with ADT/Enza by developing transcriptome-based signature scores (Figure 3) [7,33,82,87–99]. Therefore, both AR^+/hi^ LNCaP-CRPC and AR^–/lo^ LAPC9-CRPC models [33] are characterized by reduced luminal cell signature (as expected) but increased basal cell (BC), stem cell (SC), MES and neural signatures (Figure 3A–E; left). Likewise, in PCa patients subjected to short-term neoadjuvant ADT (prior to prostatectomy) [95,96], patient tumors also exhibit attenuated luminal cell gene signature but enhanced BC, SC, MES and neural gene signatures (Figure 3; right). Strikingly, the metastases in mCRPC patients who have failed long-term Enza treatment [82] manifest clear-cut lineage plasticity exhibiting much reduced luminal cell (AR signaling) signature but increased BC, SC, MES and neural gene signatures (Figure 3; right). The fact that the BC, SC, MES and neural gene signatures are coordinately and simultaneously induced in distinct experimental models and patient tumors upon ADT/Enza treatment (Figure 3) underscores that *treatment-reprogrammed, therapy-resistant PCa convergently acquires the stemness and basal, MES and neural gene expression profiles*, as well as morphological features and biological properties consistent with the 3 models proposed in Figure 1 and with the literature reports, e.g., [4,15,33,55,73,79].

There is evidence that AR^−^ PCa in treatment-naïve setting may be associated with genetic alterations such as *PTEN* loss, *TP53* mutations and *KRAS* activation (see below). In contrast, the AR^–/lo^ PCa cells that emerge from AR^+^ cells due to treatment-induced plasticity are primarily driven by epigenetic changes demonstrating the significant impact of therapeutic pressure in driving lineage plasticity and resistance [10,41,100,101]. Whether and how the initial AR^−^ cells crosstalk with AR^+^ PCa cells to influence their response to ARSIs is not well studied. How pre-existing AR^−/lo^ PCa cells differ from treatment-induced AR^−/lo^ PCa cells also awaits further investigation. As the two phenotypically identical (*i.e*., AR^−/lo^) PCa cell populations are both refractory to ADT/ARSI and a host of other therapeutic modalities [15,33], understanding the similarities and distinctions between them will be crucial for developing more effective therapeutic strategies as it underscores the need to target both genetic and epigenetic mechanisms of resistance.

**Figure 4:**
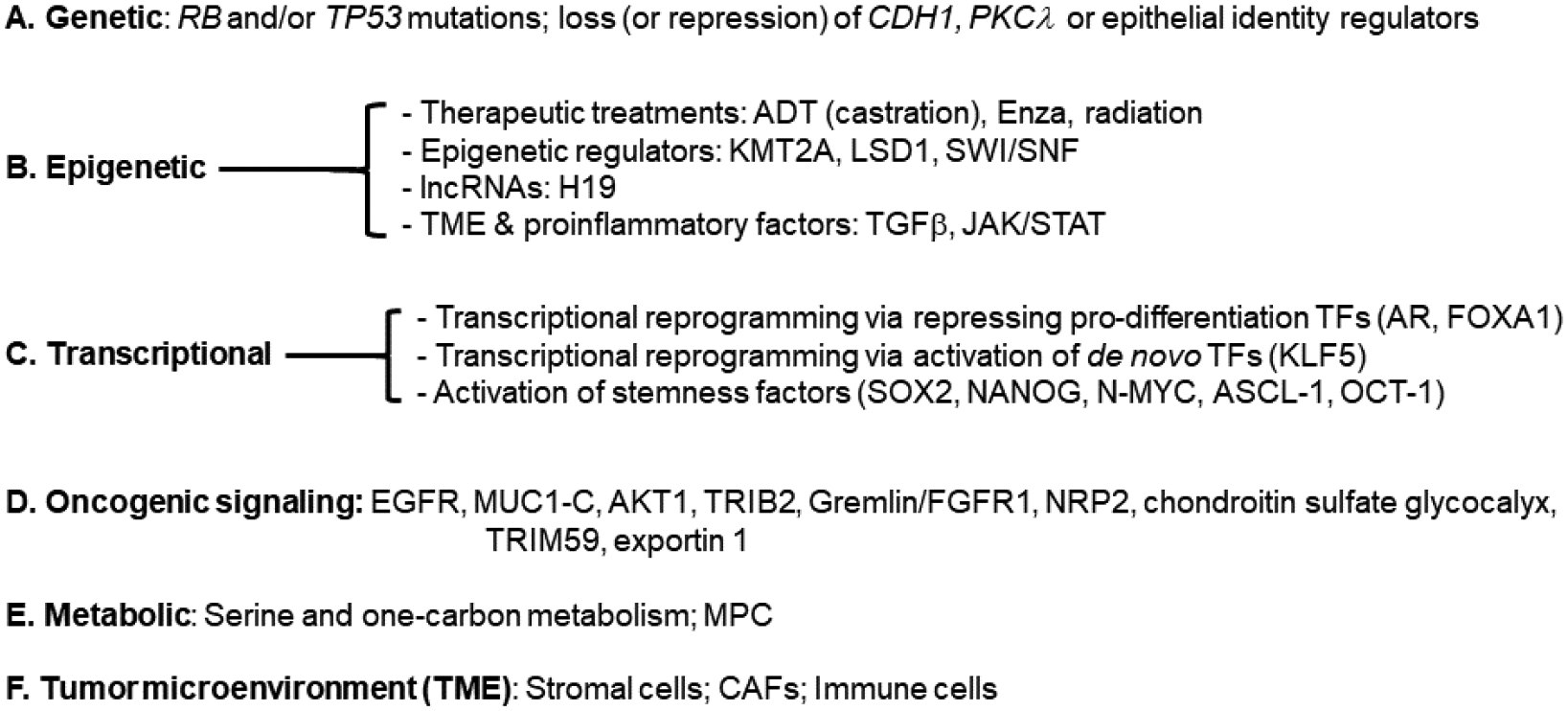
Major mechanisms driving cancer cell stemness and lineage plasticity. Shown are 6 inter-connected and inter-woven mechanisms (A–F) that have been implicated in driving up stemness and causing lineage switching in PCa development and treatment resistance. See Text for detailed discussions.

A small percentage (1–2%) of newly diagnosed PCa may present as *de novo* AR^−^ neuroendocrine PCa (NEPC), which is most frequently associated with mutations or loss of tumor suppressor genes *RB1* and/or *TP53*. As discussed above, the next-generation antiandrogens such as Enza have caused a significant increase (20–30%) in treatment-induced (or treatment-emergent) NEPC called t-NEPC or CRPC-NE, which essentially represent CRPC with significant NE features (e.g., expressing NEPC differentiation markers such as chromogranins and synaptophysin) [12,15,53]. CRPC-NE can be driven by a variety of inter-linked mechanisms involving genetic mutations/loss [6,12,15,53], epigenetic dysregulation [63,64,78,79], reactivation or upregulation of oncogenic and stemness signaling [56,66,77,83,84], transcriptional re-wiring [62,73], and metabolic reprogramming [75,76] (Figure 4; see below). Like *de novo* NEPC, CRPC-NE cells and lesions are frequently AR^–/lo^, which begs the question of relationship between loss of AR/AR signaling and acquisition of NE features. It is true that CRPC development is often accompanied by an AR switch [60] and loss of AR expression [15,33]; however, resistance to ARSIs and loss of AR expression does not necessarily lead to CRPC-NE by default [15,33,65].

It should be noted that, although ADT/Enza treatment drives significant increases in AR^−/lo^ diseases, ≥50% mCRPC remain AR^+^ (Figure 2A–F) in which canonical (pro-differentiation) AR signaling is largely inhibited by ARSIs but noncanonical AR signaling continues to function and can drive the expression of stemness-related genes and contribute to therapeutic resistance. For instance, He *et al*. [102] demonstrated that noncanonical AR addiction drives Enza resistance with a set of AR binding sites (ARBS) that have increased AR binding intensity, lack canonical androgen response elements (ARE), and are enriched in CpG islands and the binding sites for CXXC5 and TET2. Both CXXC5 and its regulated genes, including ID1, are upregulated in Enza-resistant cell lines, patient-derived xenografts (PDXs), and patient specimens. Furthermore, Enza-resistant PCa cells, organoids, xenografts, and PDXs were hypersensitive to NEO2734, a dual inhibitor of BET and CBP/p300 proteins, suggesting a potential treatment strategy [102]. WNT7B has also been nominated as a key player in noncanonical AR signaling, promoting androgen-independent growth of CRPC cells [103]. Targeting noncanonical AR pathways could be a promising therapeutic strategy to combat AR^+^ CRPC.

## 4. Drivers and regulators of lineage plasticity and therapeutic targets in PCa

**Figure 5:**
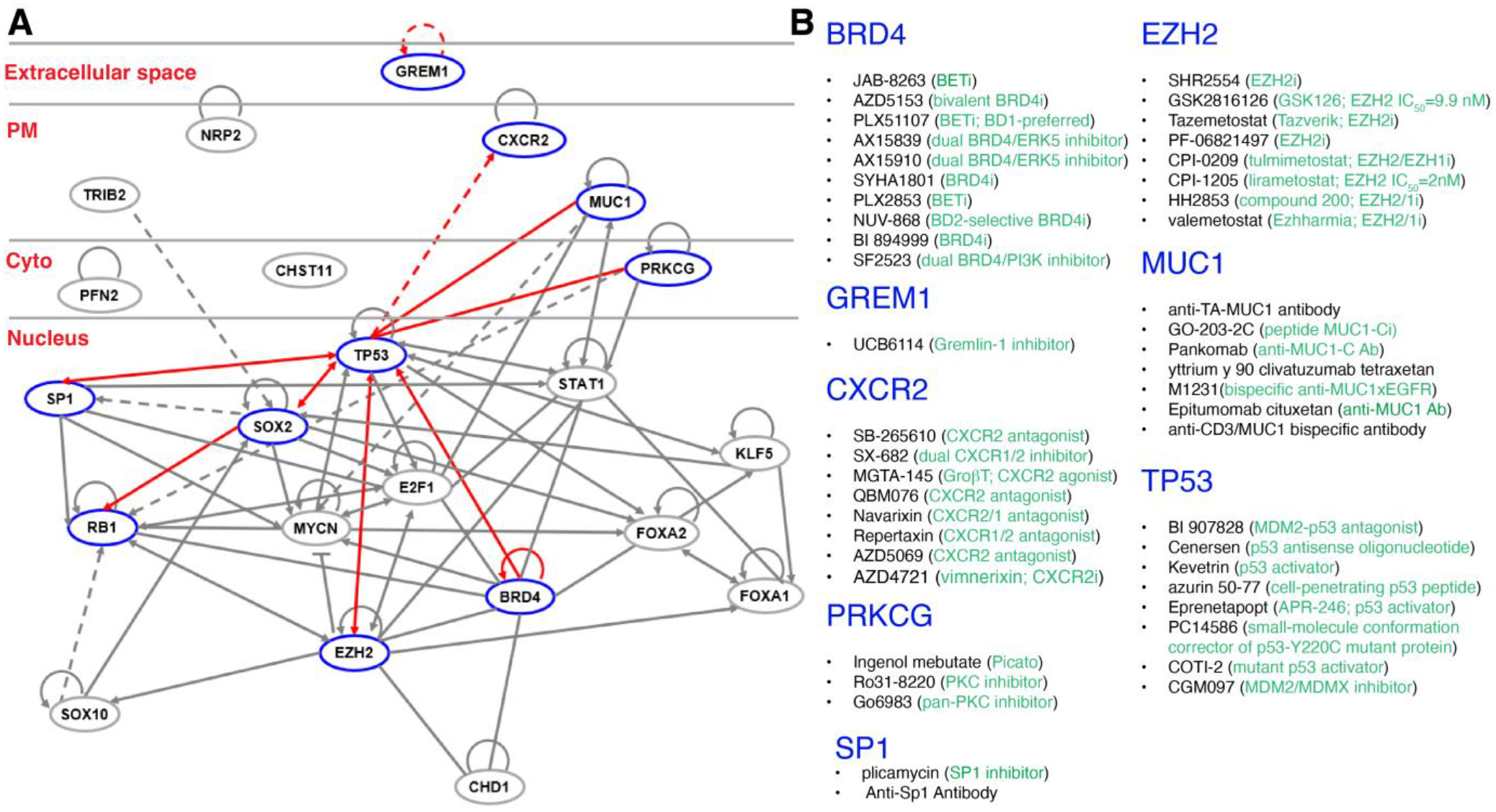
Examples of signaling networks that drive PCa stemness and lineage plasticity. (**A**) Shown are a representative gene-interaction network map in PCa, which consists of the top ranked genes with edges (lines and arrows between nodes) representing direct (solid lines) and indirect (dashed lines) interactions between molecules as supported by information in the Ingenuity knowledge base. The networks were generated using QIAGEN Ingenuity Pathway Analysis (IPA, the 2023 October Fall Release). For example, the network highlights TP53 as a central player in regulating PCa cell lineage fidelity. TP53 alterations increase from localized castration-naïve PCa (20%) to metastatic castration-naïve PCa (37%) and mCRPC (73%), implicating its role in driving resistance against ARSIs. The network also implicates *TP53* cooperation with *RB1* in regulating lineage plasticity, and their relationship edges are indicated to highlight the signaling axis and major genetic drivers of lineage plasticity. Node shapes represent functional classes of gene products (e.g., triangles for kinases, diamonds for enzymes, and ovals for transcriptional regulators or modulators). (**B**) Representative therapeutics targeting the drivers of cancer stemness and plasticity that are in clinical development or already in clinical use.

**Figure 6:**
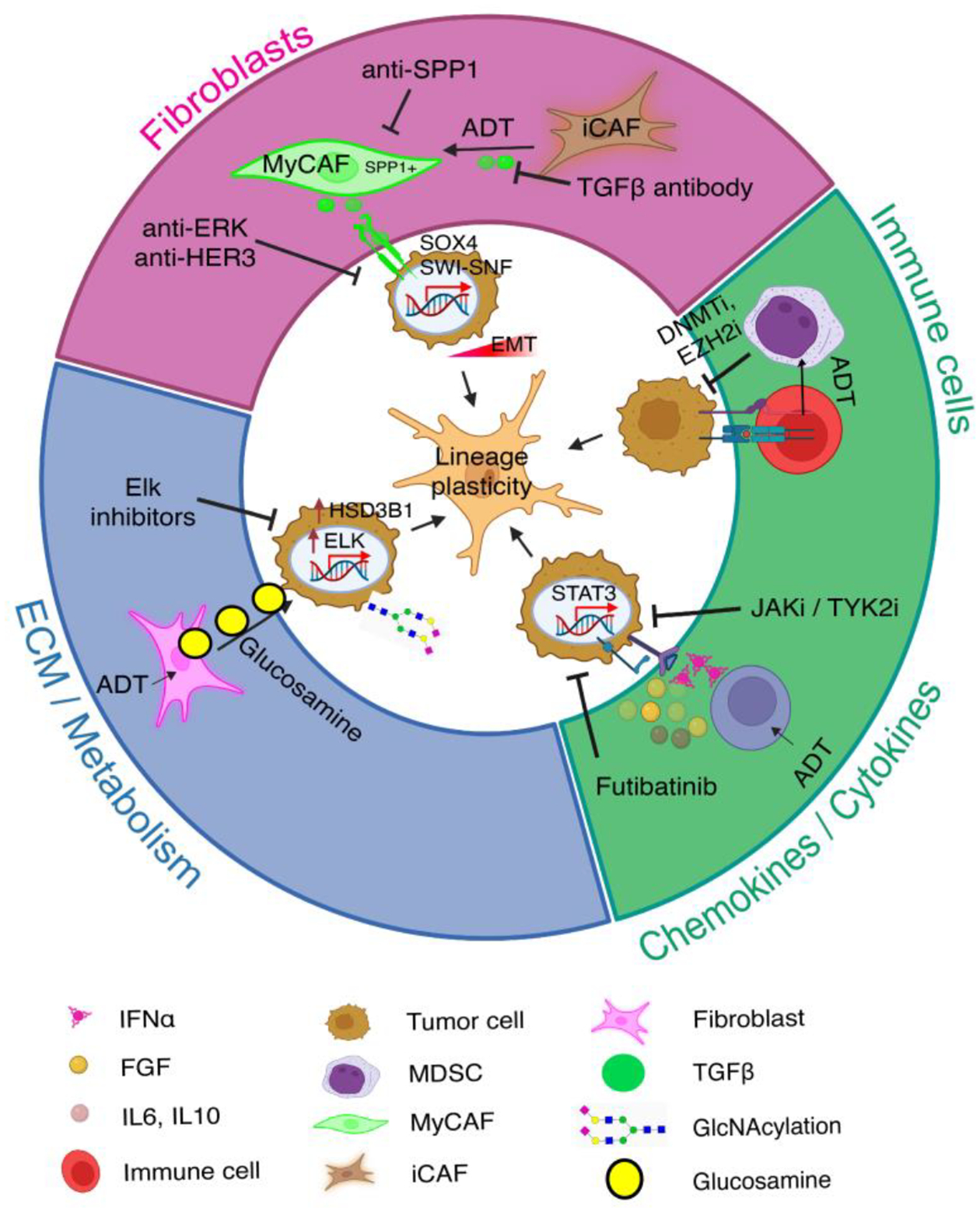
ADT-induced TME changes in regulating PCa cell plasticity and therapy resistance. ADT has profound effects on the PCa TME and PCa immune microenvironment. The fibroblasts (CAFs), and endothelial, other stromal and immune cells in the TME secrete diverse soluble and ECM signaling molecules, which may drive PCa lineage plasticity in a paracrine manner via cytokine/cytokine receptors. Additionally, metabolic products (e.g., glucosamine) secreted in ECM can also stimulate lineage plasticity via HSD3B1 activation. ADT treatment may also cause phenotypic alterations in fibroblasts, e.g., TGF-beta-driven transformation of iCAFs to SPP1^+^ MyCAFs. See Text for detailed discussions.

Lineage plasticity and stemness in PCa are driven and regulated by a spectrum of *inter-connected* and *inter-woven* mechanisms involving genetic mutations/loss, epigenetic dysregulation, heightened oncogenic and stemness signaling, transcriptional re-wiring, metabolic reprogramming, and TME changes (Figures 4–6). These mechanisms act in concert to diversify cancer cell fates and states and confer therapy resistance.

### 4.1 Genetic drivers of cancer stemness and lineage plasticity

As mentioned earlier, *de novo* NEPC most frequently involves *RB1* and/or *TP53* mutations [19]. Studies in GEMMs of PCa [4–6] have evidenced both AR heterogeneity and lineage plasticity that accompany development and progression from adenocarcinoma to NEPC driven by (co-)deletion of tumor suppressor genes *Pten*, *Rb1* and/or *p53* (Figure 4A). Thus, loss of *Pten* in prostate epithelial cells leads to slow-growing and homogeneously AR^+^ adenocarcinoma whereas co-deletion of *Pten* and *Rb1* drives formation of much more aggressive PCa that is heterogeneous in AR expression with many AR^–/lo^ PCa cells [6]. The *Pten/Rb1* co-deleted prostate tumors initially respond to castration but then develop castration-resistant disease, which, strikingly, becomes largely AR^–/lo^. When *Pten, Rb1* and *p53* are co-deleted, the mouse prostate develops *de novo* AR^−^ NEPC [6]. On the other hand, double loss of *Pten* and *p53* in prostate epithelial cells leads to tumor transformation via mesenchymal transition [4]. Lineage plasticity and CRPC development may also involve other genetic events such as *CHD1* mutations [57]. In principle, lineage plasticity caused by genetic alterations will drive innate (*versus* acquired) tumor resistance to targeted therapies.

Similarly, genetically-driven lineage plasticity may also involve dysregulated luminal epithelial regulators and loss of epithelial cell identity (Figure 4A). For instance, FOXA1 represents a ‘master’ epithelial identity TF in many epithelial organs including the lung [21], bladder [27] and prostate [50], and consequently, *FOXA1* mutations or repression of FOXA1 expression or signaling results in loss of epithelial identity, lineage promiscuity and tumorigenesis in these tissues. In PCa, FOXA1 downregulation induces TGF-beta signaling, EMT and cell motility, which can be blocked by the TGF-beta receptor I inhibitor galunisertib (LY2157299) [50]. LY2157299 treatment sensitized PCa cells to Enza, supporting a novel therapeutic strategy to control lineage switching and potentially extend clinical response to antiandrogen therapies [50]. Treatment-induced CRPC-NE may also involve reprogramming of the FOXA1 cistrome [62], *i.e*., transcriptional re-wiring (Figure 4C) of FOXA1-regulated transcriptomes. Analogous to FOXA1, *FOXA2* mutation and mis-regulation may also induce altered luminal identity, lineage plasticity and CRPC-NE via activating stem cell-related KIT signaling pathway [80]. AR represents a major regulator and enforcer of prostate epithelial identity, and prostate tumorigenesis and lineage plasticity initiated by *Pten/p53* loss [4] or *Pten/Rb1* or *Pten/Rb1/p53* co-deletion [6] entail repression of AR signaling and loss of luminal cell identity. Of interest, PCa driven by *Pten/p53* mutations also involves loss of luminal epithelial cell identity conferred by *TMPRSS2*-ERG fusion [47], the most prevalent genetic event in human PCa. CRPC-NE development involves activation of E2F1-driven cell cycle progression, which is normally repressed by AR signaling. ARSI treatment leads to repression of AR signaling, de-repression of E2F1 program and CRPC-NE transition, which can be alleviated by BET bromodomain inhibitors [64]. *CHD1*, frequently mutated in PCa, may represent another important regulator of prostate epithelial cell identity [57]. CHD1 loss was identified as a cause of antiandrogen resistance in a small hairpin RNA (shRNA) screen of 730 genes deleted in PCa. *CHD1* loss resulted in chromatin dysregulation, thereby establishing a state of transcriptional plasticity (Figure 4D) that enables the emergence of ARSI resistance. Several TFs including NR3C1, POU3F2, NR2F1, and TBX2 contributed to antiandrogen resistance associated with activation of non-luminal lineage programs [57].

### 4.2 Epigenetic regulators of cancer stemness and lineage plasticity

Epigenetic dysregulation represents a major mechanism driving stemness and lineage plasticity associated with therapy resistance (Figure 4B). ADT/Enza treatment concertedly promotes acquisition of stemness and increased cellular plasticity towards the basal/stem, MES and neural lineages in CRPC (Figure 3), which is largely mediated through epigenetic mechanisms. Treatment-naïve prostate tumors harbor a population of stem-like cancer cells called PCa stem cells (PCSCs), which are inherently resistant to ADT/Enza and behave like treatment-induced CRPC cells [15]. Recent single-cell RNA-seq (scRNA-seq) studies in patient primary PCa and CRPC have also demonstrated that CRPC-like cells preexist in untreated primary PCa and are not exclusively the result of acquired evolutionary selection during ADT [20]. This small fraction of highly plastic CRPC-like cells in hormone-naïve early PCa may undergo clonal expansion during tumor evolution towards the lethal CRPC phenotypes (Figure 1A) [20]. Experimentally, chronic exposure of LNCaP cells to different castration regimens, in 2–3 months, turned the bulk AR^+^ (and PSA^+^) cells into an AR^−^PSA^−^ phenotype, and the reprogrammed AR^−^PSA^−^ LNCaP cells increased the expression of multiple stemness and PCSC markers and were resistant to antiandrogens and docetaxel but displayed sensitivities to the BCL-2 inhibitor (ABT-199) and several kinase inhibitors [15].

Numerous chromatin (writers, readers, and binders) and DNA (methylation/demethylation) regulators have been implicated in modulating the states and lineages of cancer cells including, among others, ARID1A [28], KMT2A [30], LSD1 [49], SWI/SNF [54], CHD1 [57], and EZH2 [60]. For example, the clinical potential of reversing resistance phenotypes by redirecting cell fate was shown by modulating EZH2, which co-occupies the reprogrammed AR cistrome thereby influencing the gene networks associated with both stem cell and neuronal characteristics [60]. SWI/SNF chromatin remodeling complexes interact with different lineage-specific factors in NEPC compared to prostate adenocarcinoma, pointing to a role for SWI/SNF complexes in therapy-related lineage plasticity [54]. Overexpression of the SWI/SNF ATPase subunit SMARCA4 is associated with aggressive NE-like PCa [54]. Xu *et al*. [104] recently identified ZNF397 as essential for maintaining AR-driven luminal lineage in PCa, and its deficiency leads to a transition to a TET2-driven lineage plastic state, contributing to resistance to AR-targeted therapies.

JAK-STAT signaling has been identified as a crucial executor in promoting lineage plasticity-driven PCa resistance to ARSIs [69–71] (Figure 4B). Interestingly, JAK–STAT activation is required for the resistance of stem-like subclones expressing multilineage transcriptional programs but not subclones exclusively expressing an NE-like lineage program [69]. Lineage plasticity in PCa, initiated in an epithelial population characterized by a mixed luminal-basal phenotype, depends on elevated JAK and FGFR activity [70]. In adenocarcinoma cells, inflammatory signaling (with enrichment of response gene signatures including IFN-alpha, IFN-gamma, JAK/STAT and TNF-alpha) precedes NEPC, and JAK/STAT upregulation coincides with the onset and progression of various cellular states and phenotypes such as basal-luminal mixed lineage, EMT and transition to NEPC [70]. Moreover, STAT1 signaling-induced IFIT5 also facilitates the acquisition of stemness properties in PCa by accelerating the turnover of specific microRNAs that target CSC genes including BMI1, NANOG, and SOX2 [71]. IFN and Enza potentiate the acquisition of PCSC properties via STAT1-IFIT5 signaling [71]. These studies [69–71] suggest that targeting JAK–STAT and FGFR could inhibit PCSC phenotypes, overcome lineage plasticity and restore sensitivity to AR-targeted therapies.

Long noncoding RNAs (LncRNAs) may also regulate PCa cell plasticity through epigenetic mechanisms. For example, a high degree of association between NEPC and the imprinted lncRNA H19 has been identified by computational analysis [63]. Mechanistically, when androgen is absent, SOX2 levels increase driving the transcription of H19, which induces alterations in genome-wide DNA methylation on CpG sites, further regulating NEPC-related genes. H19 knockdown resensitizes PCa to ADT [63].

### 4.3 Transcriptional modulators of cancer stemness and lineage plasticity

Transcription is a crucial step in activation of the lineage plasticity program (Figure 4C), which, in CRPC-NE, is dependent on activation of E2F1 in concert with the BET bromodomain chromatin reader BRD4 [64] (Figure 5A). BET inhibition (BETi) blocks this E2F1/BRD4-regulated program and decreases growth of CRPC-NE models and a subset of CRPC patient tumors with high activity of this program in a BETi clinical trial [64]. Single-cell transcriptomic analysis in advanced PCa from 14 patients revealed that resistance to Enza was associated with cancer cell-intrinsic EMT and TGF-beta signaling.

As discussed earlier, ARSI-induced PCa cell plasticity and stemness is associated with downregulation of AR transcriptional activity and loss of luminal cell identity (Figure 3). RNA-seq analysis of matched biopsies prior to Enza treatment and at progression from men with mCRPC (n = 21) showed no marked Enza-induced changes in the tumor transcriptome in most patients [82]. However, 3 mCRPC patients’ progression biopsies showed evidence of lineage plasticity associated with loss of AR expression and activity and loss of luminal cell identity [82] (Figure 3, right). Notably, E2F1 and tumor stemness pathways were highly activated in the baseline biopsies in the tumors that had undergone lineage plasticity [82]. In another study, AR blockade with Enza was evinced to cause transcriptional silencing of TP53 and hence dedifferentiation of PCa cells to a hybrid epithelial and mesenchymal/stem-like state [73]. Intriguingly, this mesenchymal and stem-like PCa cell state, triggered by therapy-induced lineage plasticity, exhibited sensitivity to HER2/3 inhibition [73].

These [73,82] and many other studies vividly illustrate how shutting down AR-mediated pro-differentiation program could re-wire PCa cell transcriptional networks towards other lineages (Figure 4C). Another example is FOXA1, which normally pioneers AR chromatin binding in the prostate epithelium and thus helps maintain AR-regulated luminal cell identity. Chromatin immunoprecipitation and sequencing (ChIP-seq) identified a vast network of cis-regulatory elements (~15,000) recurrently activated in NEPC, and, strikingly, the FOXA1 cistrome was found to be reprogrammed to NE-specific regulatory elements in NEPC [62]. Ectopic expression of the NE lineage TFs ASCL1 and NKX2–1 in prostate adenocarcinoma cells pushed FOXA1 to bind to NE regulatory elements [62]. AR signaling blockade may also unleash *de novo* TFs to drive alternative cellular states (Figure 4C). For example, endocrine therapy can induce expression of the stem cell TF Krüppel-like factor 5 (KLF5), normally low or absent in PCa, leading to lineage plasticity in CRPC [61] (Figure 5A). KLF5 and AR physically interact on chromatin and drive opposing transcriptional programs, with KLF5 promoting cellular migration, anchorage-independent growth, and basal cell phenotypes [61].

PCa cell plasticity is associated with and can be induced by stemness factor reactivation or overexpression (Figure 4C; Figure 5A). For example, induced expression of a single stemness factor NANOG, which we have shown to be preferentially expressed in PCSCs and important in regulating PCSC activity [105–110], in bulk LNCaP cells gradually reprogrammed these cells into highly aggressive, stem-like and castration-resistant CRPC cells [106]. Similarly, long-term inhibition of androgen signaling induced a NE phenotype in LNCaP cells followed by ‘re-differentiation’ towards a stem-like state, with upregulation of the stemness factors NANOG and OCT4 and angiogenic factor VEGF, and downregulation of E-cadherin [55]. Mechanistically, androgen depletion induced decreased expression in AMP-activated kinase (AMPK) and stabilization of HIF-1α and forced AMPK expression in stem-like LNCaP cells restored docetaxel sensitivity [55]. SOX2 is another stemness factor implicated in driving PCa cell plasticity, metastatic progression, and therapy resistance [6,7,58] (Figure 5A). Sox2 is necessary for androgen ablation-induced NE differentiation of *Pten* null prostate adenocarcinoma as genetic ablation of *Sox2* suppressed NE differentiation [58]. However, *Sox2* deletion did not impact the castration-resistant property of castration-resistant Sox2-expressing Sca-1^+^ cells and castration-responsive Sca-1^−^ cells [58].

NEPC, CRPC-NE and small cell carcinoma of the prostate (all aggressive forms of PCa) have been shown to exhibit divergent transcriptional programs with higher expression of NANOG, SOX2 and EZH2, which promote lineage plasticity and resistance to androgen-targeting therapies [59]. Other stemness or reprogramming factors such as ASCL1 [79], N-MYC [51] and OCT1 [68] have also been implicated in driving treatment-induced PCa lineage plasticity towards NEPC. N-MYC and known AR co-factors (e.g., FOXA1 and HOXB13) overlapped, independently of AR, at genomic loci specifying the neural lineage, and histone marks specifically associated with lineage-defining genes were reprogrammed by N-MYC [51] (Figure 5A). Of interest, EZH2 inhibition has the potential to reverse N-MYC-induced suppression of epithelial lineage genes [51] (Figure 5A). OCT1 is a TF interacting with AR to enhance signaling pathways involved in PCa progression [68]. ChIP-seq studies of OCT1 in patient-derived castration-resistant AR^−^ PCa cells revealed a group of genes associated with neural precursor cell proliferation. Specifically, OCT1 targets, neural genes STNB1 and PFN2 (Figure 5A), are highly expressed in AR^−^ human PCa, and their knockdown inhibited migration of AR^−^ PCa cells and tumor growth *in vivo* [68].

### 4.4 Signaling drivers of cancer stemness and lineage plasticity

Just like loss of tumor suppressors that can elicit lineage promiscuity, activation of oncogenic signaling can also render epithelial cells to lose their differentiated phenotype (Figure 4D). For example, activated AKT1 and c-MYC are sufficient to drive lineage plasticity in human prostate luminal cells [58]. Neuropilin 2 (NRP2) is overexpressed in both *de novo* and therapy-induced NEPC, and induces NE markers, NE cell morphology and aggressive NE behavior [77] (Figure 4D; Figure 5A). NRP2 engages in reciprocal crosstalk with AR, where NRP2 is transcriptionally inhibited by AR but in turn suppresses AR signaling (by dampening AR transcriptional program) and confers Enza resistance [77]. Moreover, NRP2 physically interacts with VEGFR2 through the intracellular SEA domain to activate STAT3 and SOX2 driving NEPC differentiation and growth [77]. FGFR1 is a driver of lineage plasticity and castration resistance, and its non-canonical ligand Gremlin1 (GREM1) is negatively regulated by AR and highly expressed in CRPC following ADT, facilitating the development of castration resistance in PCa cells [72] (Figure 5A). A recently developed anti-Gremlin1 therapeutic antibody demonstrated synergistic effect with ADT in inhibiting CRPC growth [72] (Figure 5B). Pseudokinase Tribbles 2 (TRIB2) is overexpressed in Enza-resistant PCa cells and drives ARSI resistance by promoting cellular plasticity and lineage switching [67] (Figure 5A). TRIB2 downregulates AR and cytokeratin 8 but upregulates the neuronal TF BRN2 and the stemness factor SOX2 to induce NE characteristics, and, notably, Enza resistance is lost upon inhibition of TRIB2 [67].

MUC1-C oncoprotein is overexpressed CRPC and NEPC and represents a novel effector of lineage plasticity driving progression to NEPC [56] (Figure 4D; Figure 5A). Upregulation of MUC1-C in androgen-dependent PCa cells represses AR signaling and induces the neural TF BRN2 and NE differentiation [56]. Moreover, MUC1-C suppresses the p53 pathway and drives stemness by inducing OCT4, SOX2, KLF4 and MYC pluripotency factors. Targeting MUC1-C inhibited PCa self-renewal and tumorigenicity, suggesting a potential therapeutic approach for CRPC and NEPC [56] (Figure 5B). A reactive chondroitin sulfate (CS) glycocalyx supports adaptive survival and treatment resistance after AR pathway inhibition, and inhibition of tumor cell CS glycocalyx delays CRPC progression [81]. AR directly represses transcription of the 4-O-sulfotransferase gene *CHST11* under basal androgen conditions, maintaining steady-state CS in prostate adenocarcinomas. When AR signaling is inhibited, CHST11 expression is upregulated, resulting in elevated 4-O-sulfated chondroitin levels [81] (Figure 5A).

Recent research has identified a non-neuroendocrine small cell-like PCa (non-NE SCLPC) subtype that pre-exists in primary PCa with admixed histology [111]. This subtype, characterized by AR-expressing but lacking neuroendocrine features, exhibits morphological and molecular characteristics of small cell carcinoma. These SCLPC cells are clinically aggressive, displaying low AR activity but high stemness and proliferation. Molecular analysis showed that protein translation and the TF SP1 are critical drivers of the SCLPC phenotype (Figure 5A). Targeting SP1 with specific inhibitors, such as plicamycin, has shown efficacy in suppressing CRPC growth *in vivo* (Figure 5B). Additionally, homoharringtonine, an FDA-approved translation elongation inhibitor, has been effective in impeding CRPC progression in preclinical models. This research suggests that SP1 and translation elongation are actionable therapeutic targets for combating ARSI resistance [111].

Finally, functional studies revealed CXCR2 to be a driver of the NE phenotype, including loss of AR expression, lineage plasticity and resistance to hormonal therapy [34] (Figure 5A). CXCR2-driven NE cells are critical for the tumor microenvironment by providing a survival niche for AR^+^ luminal cells; consequently, combination of CXCR2 inhibition (Figure 5B) and AR targeting effectively inhibited lineage plasticity and NE transition in mouse xenograft models [34].

### 4.5 Metabolic reprogramming associated with cancer stemness and lineage plasticity

As mentioned in the beginning of this section, treatment-induced cancer stemness and lineage plasticity invoke and necessitate several inter-connected mechanisms including metabolic adaptation and reprogramming (Figure 4E) [112]. For example, SOX2, the stemness factor that helps drive lineage switching [6,7,58] (Figure 5A), also promotes changes in multiple metabolic pathways including increased glycolysis and glycolytic capacity, increased basal and maximal oxidative respiration, and increased spare respiratory capacity [75]. Metabolic rewiring can in principle act as a starter for increased cellular plasticity leading to antiandrogen resistance through lineage switching [112]. In PCa, this principle of metabolism-associated cellular plasticity was demonstrated to be enabled by the loss of mitochondrial pyruvate carrier (MPC), the gatekeeper for mitochondrial pyruvate influx [76]. As a result, MPC restoration and overexpression inhibited treatment-induced transition of hormone-sensitive PCa to lethal CRPC-NE [76].

### 4.6 Role of TME in driving PCa cell plasticity and therapy resistance

Various TME components, such as stromal cells, immune cells, and physicochemical factors, dynamically interact with each other and with cancer cells, aiding PCa cell adaptation and survival under therapeutic pressure (Figure 6).

#### 4.6.1 Induction of PCa cell mesenchymal plasticity by EMT signals

As presented in Figure 1, induction of EMT or mesenchymal transformation represents one of the mechanisms driving cancer cell lineage switching. A recent study investigated whether an increase in EMT activity was also associated with gene expression programs involved in lineage plasticity, and authors found that the stem-like and NE lineages are largely correlated with the PCa cell EMT level [113]. These findings are analogous to our data showing that ADT/Enza convergently drive up the alternative (*i.e*., epithelial to mesenchymal and basal-/stem-/neural-like) cell fates in PCa adenocarcinoma cells (Figure 3). Their study uncovered some new EMT-inducing spatial interactions among PCa cells, fibroblasts and endothelial cells [113].

#### 4.6.2 Stromal cell reprogramming and cancer-associated fibroblasts (CAFs) in mediating ARSI resistance

Classic tissue recombination experiments showed that during organogenesis AR^+^ mesenchyme cells could promote AR^−^ epithelial cells to grow and generate prostate tissues through paracrine signals. Stromal cells, including CAFs, are key components of the TME. CAFs in the PCa TME may contribute to therapy resistance by enhancing cancer cell survival, proliferation, and metastasis via secreting growth factors, cytokines, and extracellular matrix components and by promoting angiogenesis, immune evasion, and EMT. Vice versa, ADT and antiandrogen treatments may also reprogram stromal cells, fostering CRPC. For instance, Wang *et al*. recently showed that ADT treatment promoted transformation of inflammatory CAFs (iCAFs) to SPP1^+^ myofibroblastic CAFs (MyCAFs), which in turn interacted with PCa cells and induced EMT in PCa cells via TGF-beta/SWI-SNF signaling [114] (Figure 6). Depletion of the SPP1^+^ MyCAFs in genetic mouse models prolonged the lifespan of the mice and reduced prostate tumor volume [114].

#### 4.6.3 Impact of immune cells and physicochemical factors in the TME on PCa cell plasticity

Immune cells and physicochemical factors in the TME influence tumor cell behavior through epigenetic modifications, such as DNA methylation and histone modification (Figure 6). These changes can reprogram cancer cells into more aggressive and therapy-resistant phenotypes. Epigenetic regulation of immune cells, like CD8^+^ T cells, may in turn impact their cytotoxic activities and the immune response against tumor cells, affecting cancer progression and therapy resistance [104,115]. Radiotherapy can also modulate the TME by inhibiting CD8^+^ T cell infiltration, contributing to radio-resistance [116]. Proinflammatory cytokines and other factors in the TME may also regulate cancer cell stemness and plasticity via epigenetic, transcriptional and signaling mechanisms, as exemplified in Figure 6. Thus, as also discussed above (Figure 4B), lineage plasticity in PCa cells can be induced by cytokines and JAK/STAT signaling, which also promote transcription of genes involved in immune cell division, survival, activation, and recruitment [69–71].

#### 4.6.4 Metabolic and extracellular matrix (ECM) changes in the TME drive PCa plasticity

Metabolic alterations in the TME, such as hypoxia, are known to promote cancer cell adaptability and cancer stemness [15]. Two recent studies from Dr. Sharifi lab further illustrate the importance of the metabolic and ECM alterations in the TME in driving PCa therapy resistance [117,118]. In PCa receiving ADT, CAFs in the TME secrete glucosamine, a proteoglycan abundant in the ECM, which subsequently promoted O-GlcNAcylation as well as increased expression of nuclear TF Elk1 in PCa cells. Increased Elk1 in turn induced 3 beta-HSD1 (HSD3B1) transcription, leading to *de novo* intratumoral androgen synthesis to overcome castration effects [117] (Figure 6). As a result, Elk1 inhibitors could dampen the CAF-originated, glucosamine-initiated intracrine androgen biosynthesis using extragonadal substrates and inhibit CRPC [117] (Figure 6). A follow-up study from the same group revealed that 3βHSD1, the rate-limiting enzyme in catalyzing the intra-tumoral androgen synthesis from non-testicular substrates such as DHEA (dehydroepiandrosterone), becomes stabilized by hypoxia via repressing autophagy-related genes [118].

#### 4.6.5 Therapeutic targeting of the TME-regulated mechanisms of PCa cell plasticity and therapy resistance

Understanding TME and lineage plasticity interactions opens new therapeutic avenues. As depicted in Figure 6, targeting TME-regulated lineage plasticity in PCa cells could enhance treatment effectiveness and reduce resistance against ARSIs. For example, stromal cells such as CAFs in PCa TME promote therapy resistance and CRPC development by triggering PCa cell EMT through secreting cytokines such as TGF-beta, by reprogramming CAFs themselves from iCAFs to SPP1^+^ MyCAFs, or by secreting the ECM proteoglycan glucosamine to enhance intracrine androgen production (Figure 6). On the other hand, androgen blockade through ADT and antiandrogens may promote the production of soluble cytokines/chemokines which can in turn drive PCa cell plasticity by activating JAK/STAT signaling (Figure 6). Meanwhile, ADT/antiandrogens may promote interactions between cytotoxic T cells and immune-suppressive MDSCs (myeloid-derived suppressor cells) leading to immune evasion of PCa cells (Figure 6). Intervening each of these newly discovered signaling pathways in the TME may help curb PCa lineage plasticity, extend the therapeutic window of ADT/antiandrogens, and inhibit CRPC development (Figure 6).

## 5. Conclusions and perspectives

It is easy to understand that molecularly targeted therapies mainly eliminate the cancer cell population expressing the therapeutic target. It takes some efforts to appreciate that intrinsic cancer cell heterogeneity in target expression may allow target-negative cancer cells, which often behave as immature precursor cells and possess CSC properties, to differentiate into target-positive daughters and repopulate the recurrent tumor. Moreover, treatment may elicit cellular and lineage plasticity pushing target-positive therapy-sensitive cells towards a target-negative and therapy-insensitive state. To achieve long-lasting therapeutic efficacy, we need to develop rational and novel combinatorial protocols to both target cancer cell heterogeneity and curb induced cancer plasticity. Take PCa, a host of molecular regulators and drivers of cancer stemness and lineage plasticity, in both PCa cells proper (Figure 5) and the PCa TME (Figure 6), has been reported. Examples include extracellular ligand (e.g., GREM1 [72]) to receptors on the plasma member (e.g., CXCR2 [35], NRP2 [77] and MUC1-C [56]) to signal transducers in the cytoplasm (e.g., PFN2 [68], CHST11 [81]) and finally, to a large repertoire of nuclear factors including TFs (RB1, TP53, E2F1, STAT [69–71], MYCN [51]), luminal epithelial cell determinants (FOXA1/A2 [21,27,50,62,80]), stemness regulators (SOX2 [58,75], KLF5 [61]) and epigenetic modifiers (CHD1 [57], EZH2, BRD4 [64] (Figure 5A). Importantly, numerous therapeutics (small-molecule drugs, antibodies, cell-penetrating peptides, antisense oligonucleotides, *etc*.) that target the drivers of stemness and lineage plasticity have been developed, many of which are in clinical development or already in clinical use (Figure 5B). It is conceivable that the combined use of ARSIs with the stemness- and plasticity-targeting drugs such as BRD4 and EZH2 inhibitors or with TP53 activators (Figure 5B) may likely achieve better and more enduring therapeutic efficacy than ARSIs alone.

## Abbreviations

The following abbreviations are used in this manuscript:

[^18^F]-FDG: [18F]-labeled fluoro-2-D-deoxyglucose
[^18^F]-FDHT: [18F]-labeled fluorodihydrotestosterone
Abi: abiraterone acetate
ADT: androgen deprivation therapy
ALL: acute lymphoblastic leukemia
AMP: amplification
AMPK: AMP-activated kinase
AR: androgen receptor
ARBS: AR binding site
ARE: androgen response element
ARSI: androgen receptor signaling inhibitor
BC: basal cell
BETi: BET inhibitor
CAF: cancer-associated fibroblast
ChIP-seq: chromatin immunoprecipitation and sequencing
CRPC: castration-resistant prostate cancer
CS: chondroitin sulfate
CSC: cancer stem cell
Cyto: cytoplasm
DNMTi: DNA methyltransferase inhibitor
ECM: extracellular matrix
EMT: epithelial-to-mesenchymal transition
Enza: enzalutamide
ER: estrogen receptor
EZH2i: Enhancer of Zeste Homolog 2 (EZH2) inhibitor
GEMM: genetically engineered mouse model
HCC: hepatocellular carcinoma
iCAF: inflammatory cancer-associated fibroblast
IP: Immunoprecipitation
IPA: Ingenuity Pathway Analysis
JAKi: Janus kinase (JAK) inhibitor
LncRNA: Long noncoding RNA
LUAD: lung adenocarcinoma
LumA: luminal A
LumB: luminal B
mCRPC: metastatic castration-resistant prostate cancer
MDSC: myeloid-derived suppressor cell
MES: mesenchymal
mHSPC: metastatic hormone-sensitive prostate cancer
MPC: mitochondrial pyruvate carrier
MSigDB: Molecular Signatures Database
MSK-IMPACT: Memorial Sloan Kettering-Integrated Mutation Profiling of Actionable Cancer Targets
MyCAF: myofibroblastic cancer-associated fibroblast
NE: neuroendocrine
NEPC: neuroendocrine prostate cancer
NSCLC: non-small cell lung cancer
PAM50: Prediction Analysis of Microarray using the 50-gene classifier (The Prosigna Breast Cancer Prognostic Gene Signature Assay)
PCa: prostate cancer
PCS: prostate cancer classification system
PCSC: prostate cancer stem cell
PDAC: pancreatic ductal adenocarcinoma
PDX: patient-derived xenograft
PET/CT: positron emission tomography/computed tomography
PM: plasma membrane
Pri-PCa: primary prostate cancer
PSA: prostate-specific antigen
RNA-seq: RNA sequencing
SC: stem cell
SCLC: small cell lung cancer
SCLPC: small cell-like prostate cancer
scRNA-seq: single-cell RNA sequencing
shRNA: small hairpin RNA
ssGSEA: single sample gene set enrichment analysis
SU2C: stand up to Cancer
t-NEPC: treatment-induced (or treatment-emergent) neuroendocrine prostate cancer
TF: transcription factor
TKI: tyrosine kinase inhibitor
TMA: tissue microarray
TME: tumor microenvironment
TYK2i: Tyrosine kinase 2 (TYK2) inhibitor
WM: whole-mount
